# The diagnostic value of morphological features of fat deposition of sacroiliac joint steatosis in axial spondyloarthritis

**DOI:** 10.3389/fmed.2023.1218834

**Published:** 2023-08-23

**Authors:** Jiaoshi Zhao, Churong Lin, Dong Liu, Budian Liu, Qilong Chen, Jieruo Gu

**Affiliations:** ^1^Department of Rheumatology and Immunology, Third Affiliated Hospital of Sun Yat-sen University, Guangzhou, China; ^2^Department of Radiology, Third Affiliated Hospital of Sun Yat-sen University, Guangzhou, China

**Keywords:** morphological features, fat deposition, sacroiliac joint steatosis, axial spondyloarthritis, diagnostic value, chemical shift-encoded MRI

## Abstract

**Background:**

Findings of fatty lesions in the context of other imaging manifestations, especially bone marrow edema and erosions can effectively assist in the diagnosis of axSpA. Chemical shift-encoded MRI is a sequence which allows for the quantification of fat signal and has been applied in the imaging evaluation of the SIJ in axSpA. The objective of this study was to investigate the diagnostic performance of morphological features of fatty lesions visualized by CSE-MRI in the imaging evaluation of SIJ in axSpA.

**Methods:**

Fatty lesions with morphological features (subchondral, homogeneity and distinct border) were assessed and recorded as a binary variable in each quadrant of the SIJ. Sensitivity, specificity, positive predictive value (PPV) and negative predictive value (NPV) were calculated for different morphological features as well as the anatomical distribution in patients with nr-axSpA and r-axSpA. T1-weighted images and CSE-MRI fat fraction maps were directly compared in the recognition of different morphological features.

**Results:**

Eighty-two patients [non-SpA (*n* = 21), nr-axSpA (*n* = 23), r-axSpA (*n* = 38)] with lower back pain (LBP) were enrolled. Presence of the three morphological features of fatty lesions had a specificity of 90.48% in axSpA. The sensitivities of being subchondral, homogeneity and distinct border were 52.17, 39.13 and 39.13% in nr-axSpA on T1-weighted images. For patients with r-axSpA, the sensitivities reached 86.84, 76.32 and 57.89%. No significant difference was found in the distribution of fatty lesions between T1-weighted images and CSE-MRI. However, CSE-MRI fat fraction maps could detect significantly more fatty lesions with homogeneity (*p* = 0.0412) and distinct border (*p* = 0.0159) than T1-weighted images in the sacroiliac joint, but not subchondral lesions (*p* = 0.6831).

**Conclusion:**

The homogeneity and distinct border are more relevant for the diagnosis of axSpA. Moreover, CSE-MRI could detect more typical morphological features of fatty lesions than T1-weighted images in showing these two features. The presence of all three features was more likely to be indicative of axSpA.

## Introduction

As per the standardized definitions of MRI lesions in the sacroiliac joint of patients with axial spondyloarthritis by the ASAS MRI working group, fat metaplasia belongs in the group of structural lesions (1). Although fatty lesion is a common finding on the T1-weighted images, its relevance to the diagnosis of axSpA has been widely debated (2). According to a large population-based cohort study investigating frequency of MRI changes, fatty lesion is also very common in healthy individuals, hence its diminished specificity (3). Findings of fatty lesions are advised to be interpreted in the context of other imaging manifestations, especially bone marrow edema and erosion (4).

Three morphological features of fat infiltration have been defined so as to facilitate better characterization of such fatty lesions: (1) a distinct border around the fatty lesion; (2) homogeneity of the increased T1-weighted signal; (3) proximity of the fatty lesion to subchondral bone of SIJ (1). Previous studies investigating the diagnostic utility of these features concluded that SIJ fatty lesion *per se* does not facilitate the recognition of nr-axSpA, while distinct border or homogeneity showed small to moderate diagnostic utility, which was strongly associated with concomitant bone marrow edema and erosion (5, 6).

Chemical shift-encoded MRI is a sequence which allows for the quantification of fat signal (7–9) and has been applied in the imaging evaluation of the SIJ in axSpA (10). Previous studies demonstrated that the SIJ fat fraction is significantly higher in patients with axSpA, especially in r-axSpA (2, 11). Utilizing the quantification of fat fraction, CSE-MRI could generate fat fraction maps, which could be employed as a promising tool to visualize the morphological features of fatty lesions. This study aims to investigate the diagnostic utility of morphological features of fatty lesions visualized by CSE-MRI in the imaging evaluation of SIJ in axSpA.

## Methods

### Subjects

Participants complaining of lower back pain (LBP) who visited the outpatient clinic of the Department of Rheumatology of the Third Affiliated Hospital of Sun Yat-sen University from June 1, 2020 to January 20, 2022 were enrolled in this study. The inclusion criteria consisted of: (1) Chronic lower back pain ≥3 months; (2) Aged ≤50 years. All participants were required to meet: (a) BMI ≤30 kg/m2; (b) No history of other rheumatic immune disorders; (c) no history of malignant neoplasms; (d) no contraindications for MRI. Clinical data such as age, sex, age of onset, duration of disease, past medical history, smoking history, history of medication was collected with questionnaires. Disease activity was assessed using the Bath Ankylosing Spondylitis Disease Activity Index (BASDAI) and the Ankylosing Spondylitis Disease Activity Score – CRP (ASDAS-CRP).

The diagnosis of each participant was determined by the expert panel, which consisted of two rheumatologists and one radiologist. Based on the clinical information and the pelvic radiographs, study subjects were classified as non-SpA (*n* = 21), nr-axSpA (*n* = 23), r-axSpA (*n* = 38). Patients determined as non-SpA were regarded as the control group. Grading of the radiographic sacroiliitis was conducted by the radiologist according to the modified New York criteria. Patients with previous or current treatment with biologics were excluded from this study. This study was conducted abiding by the Declaration of Helsinki and was approved by the Ethics committee of the Third Affiliated Hospital of Sun Yat-sen University. Informed consent was obtained from all the participants.

### Evaluation of MR images

MRI images were acquired on 3.0 T SignaTM Architect (GE). T1 weighted, T1 fat-saturated (T1-FS) sequences and T2 fat-saturated (T2-FS) sequences in a semi-coronal orientation and T2 FS sequences in a semi-axial orientation were obtained. Scoring of fat deposition and erosions was based on T1 weighted images. The SIJ MRI scans were evaluated independently by two observers in a random order on the workstation of the institution. All the scans were evaluated by both observers twice in order to assess the intra-reader agreement. Fatty lesions with various morphological features were determined when both observers unanimously agreed on the presence of the lesions.

The following morphological features were assessed across the MRI slices: (1) Subchondral: whether the fatty lesion is located in the subchondral area under the cartilage; (2) Homogeneity: whether the signal within the fatty lesion is homogeneous; (3) Distinct border: whether the fatty lesion has a distinct contour. Assessment of the morphological features were recorded as a binary variable in each quadrant of the SIJ. Prior to the imaging evaluation, a training SIJ MRI set was established in order to reach consensus between both observers.

### Statistical analysis

Software packages R version 3.6.3 (The R Project for Statistical Computing, Vienna, Austria) was used for all statistical analysis. Characteristics of the study subjects were summarized using descriptive statistics. The mean ± SD or the median and IQR were presented according to the distribution of the individual characteristics. We computed the proportions of patients with fatty lesions in at least 1, 2, 3, 4 quadrants of SIJ in different groups. Frequencies of the three morphological features were also calculated in each group.

Sensitivity, specificity, positive predictive value (PPV) and negative predictive value (NPV) were calculated for different morphological features as well as the anatomical distribution. Such analyses were conducted in patients with nr-axSpA and r-axSpA separately. T1-weighted images and CSE-MRI fat fraction maps were directly compared in the recognition of different morphological features. Images were defined as T1(+)CSE-MRI(+), T1(+)CSE-MRI(+), T1(−)CSE-MRI(+), T1(−)CSE-MRI(−) based on the features seen on the two sequences. The McNemar’s test was used to analyze whether there was a difference between the two sequences. Unweighted Cohen’s Kappa was calculated to compare the inter-reader and intra-reader agreement of the T1-weighted images and CSE-MRI fat fraction maps. A *p* value <0.05 was considered significant in all analyses.

## Results

### Demographic characteristics

This study included 82 patients with lower back pain. 21 patients were classified as non-SpA, while 23 were nr-axSpA and 38 were r-axSpA. Demographic and clinical characteristics were listed in Table 1. Compared with non-SpA patients, a significantly higher proportion of patients with axSpA were male. Patients with r-axSpA had a significantly longer disease duration compared with non-SpA and nr-axSpA (median 5.5 years vs. 2 years and 2 years, *p* < 0,001). Patients with r-axSpA also had a higher disease activity than nr-axSpA, as indicated by the ASDAS-CRP (median 2.09 vs. 1.27, *p* = 0.008).

**Table 1:** Demographic data and clinical characteristics.

	non-SpA (*n* = 21)	nr-axSpA (*n* = 23)	r-axSpA (*n* = 38)	*p* value
Age years(mean ± SD)	31.4 ± 7.82	28.2 ± 10.4	32.0 ± 9.41	0.290
Male, *n* (%)	8 (38.10%)	18 (78.26%)	27 (71.05%)	0,011*
Duration of illness (years) Median [interquartile range]	2 [1–3]	2 [0.5–3.5]	5.5 [3.25–9]	<0.001*
BMI(kg/m^2^)， Median [interquartile range]	21.4 [18.7–22.9]	21.5 [20.3–22.6]	22.3 [19.8–25.4]	0.411
Smoking, *n* (%)	1 (4.76%)	3 (13.04%)	18 (47.37%)	0.018*
HLA-B27(+), *n* (%)	5 (23.81%)	18 (78.26%)	36 (94.74%)	<0.001*
ASDAS-CRP， Median [interquartile range]		1.27 [0.92–2.03]	2.09 [1.63–2.87]	0.008*
BASDAI， Median [interquartile range]		1 [0.4–1.95]	1 [0.4–2.88]	0.536
bDMARDs, *n* (%)		5 (21.74%)	15 (39.47%)	0.251

*p < 0.05.

### Distribution of fat deposition in SIJ and its morphological features

On the T1-weighted images, the frequencies of being subchondral, homogeneity and distinct border in non-SpA patients were 23.81, 9.52 and 14.29% respectively, while the frequencies were 52.17, 39.13, 39.13% in nr-axSpA and 86.84, 76.32 and 57.89% in r-axSpA.

On the CSE-MRI fat fraction maps, the frequencies of such morphological features were 23.81, 14.29, 14.29% in non-SpA patients. For nr-axSpA patients with r-axSpA, the frequencies were 51.17, 47.83, 52.17 and 92.11%, 84.21, 73.68%, respectively.

The proportions of non-SpA patients with at least 1, 2 and 3 morphological features seen on T1-weighted images were 23.81, 14.29 and 9.52%, respectively, while in nr-axSpA and r-axSpA patients, the proportions were 65.22, 39.13, 26.09 and 89.47%, 76.32, 55.26%, respectively. In contrast, on CSE-MRI images, the proportions of non-SpA patients were 23.81, 14.29 and 14.29%. The proportions were 65.22, 52.17, 34.78 and 92.11%, 84.21 73.68% in nr-axSpA and r-axSpA patients, respectively (Shown in Table 2).

**Table 2:** Frequencies of fatty lesions with different anatomical distributions and morphological features on T1-weighted images and CSE-MRI fat fraction maps.

	T1			CSE-MRI fat fraction map		
	non-SpA	nr-axSpA	r-axSpA	non-SpA	nr-axSpA	r-axSpA
Fat lesion						
At least in 1 quadrant	8 (38.10%)	17 (73.91%)	36 (94.73%)	8 (38.10%)	18 (78.26%)	37 (97.37%)
At least in 2 quadrants	7 (33.33%)	15 (65.22%)	34 (89.47%)	7 (33.33%)	16 (69.57%)	35 (92.11%)
At least in 3 quadrants	1 (4.76%)	12 (52.17%)	30 (78.95%)	1 (4.76%)	12 (52.17%)	31 (81.58%)
At least in 4 quadrants	1 (4.76%)	11 (47.83%)	30 (78.95%)	1 (4.76%)	11 (47.83%)	31 (81.58%)
Characteristics of fat lesion						
Subchondral	5 (23.81%)	12 (52.17%)	33 (86.84%)	5 (23.81%)	12 (52.17%)	35 (92.11%)
Homogeneity	2 (9.52%)	9 (39.13%)	29 (76.32%)	3 (14.29%)	11 (47.83%)	32 (84.21%)
Distinct border	3 (14.29%)	9 (39.13%)	22 (57.89%)	3 (14.29%)	12 (52.17%)	28 (73.68%)
With at least 1 feature	5 (23.81%)	15 (65.22%)	34 (89.47%)	5 (23.81%)	15 (65.22%)	35 (92.11%)
With at least 2 features	3 (14.29%)	9 (39.13%)	29 (76.32%)	3 (14.29%)	12 (52.17%)	32 (84.21%)
With at least 3 features	2 (9.52%)	6 (26.09%)	21 (55.26%)	3 (14.29%)	8 (34.78%)	28 (73.68%)

### Diagnostic utilities of morphological features

The sensitivity, specificity, PPV and NPV of various morphological features pertinent to fat deposition on MRI in the diagnosis of nr-axSpA and r-axSpA were shown in Tables 3, 4. The sensitivities of being subchondral, homogeneity and distinct border were modest in nr-axSpA on T1-weighted images, reaching 52.17, 39.13 and 39.13%, respectively. For patients with r-axSpA, the sensitivities reached 86.84, 76.32 and 57.89%. Fatty lesions with all three features could only be observed in 26.09% of the nr-SpA patients, while such lesions could be found in 55.26% of r-axSpA patients. However, such typical lesions could be very specific to axSpA, with a specificity of 90.48%.

**Table 3:** Diagnostic utilities of morphological features pertinent to fat metaplasia in patients with nr-axSpA.

	Sensitivity		Specificity		PPV		NPV	
	T1	CSE-MRI	T1	CSE-MRI	T1	CSE-MRI	T1	CSE-MRI
Fat lesion								
At least in 1 quadrant	73.91%	78.26%	61.90%	61.90%	68.00%	69.23%	68.42%	72.22%
At least in 2 quadrants	65.22%	69.57%	66.67%	66.67%	68.18%	69.57%	63.64%	66.67%
At least in 3 quadrants	52.17%	52.17%	95.24%	95.24%	92.31%	92.31%	64.52%	64.52%
At least in 4 quadrants	47.83%	47.83%	95.24%	95.24%	91.67%	91.67%	62.50%	62.50%
Characteristics of fat lesion								
Subchondral	52.17%	52.17%	76.19%	76.19%	70.59%	70.59%	59.26%	59.26%
Homogeneity	39.13%	47.83%	90.48%	85.71%	81.82%	78.57%	57.58%	60.00%
Distinct border	39.13%	52.17%	85.71%	85.71%	75.00%	80.00%	56.25%	62.07%
With at least 1 feature	65.22%	65.22%	76.19%	76.19%	75.00%	75.00%	66.67%	66.67%
With at least 2 features	39.13%	52.17%	85.71%	85.71%	75.00%	80.00%	56.25%	62.07%
With at least 3 features	26.09%	34.78%	90.48%	85.71%	75.00%	72.73%	52.78%	54.55%

**Table 4:** Diagnostic utilities of morphological features pertinent to fat metaplasia in patients with r-axSpA.

	Sensitivity		Specificity		PPV		NPV	
	T1	IDEAL	T1	IDEAL	T1	IDEAL	T1	IDEAL
Fat lesion								
At least in 1 quadrant	94.74%	97.37%	61.90%	61.90%	81.82%	82.22%	86.67%	92.86%
At least in 2 quadrants	89.47%	92.11%	66.67%	66.67%	82.93%	83.33%	77.78%	82.35%
At least in 3 quadrants	78.95%	81.58%	95.24%	95.24%	96.77%	96.88%	71.43%	74.07%
At least in 4 quadrants	78.95%	81.58%	95.24%	95.24%	96.77%	96.88%	71.43%	74.07%
Characteristics of fat lesion								
Subchondral	86.84%	92.11%	76.19%	76.19%	86.84%	87.50%	76.19%	84.21%
Homogeneity	76.32%	84.21%	90.48%	85.71%	93.55%	91.43%	67.86%	75.00%
Distinct border	57.89%	73.68%	85.71%	85.71%	88.00%	90.32%	52.94%	64.29%
With at least 1 feature	89.47%	92.11%	76.19%	76.19%	87.18%	87.50%	80.00%	84.21%
With at least 2 features	76.32%	84.21%	85.71%	85.71%	90.63%	91.43%	66.67%	75.00%
With at least 3 features	55.26%	73.68%	90.48%	85.71%	91.30%	90.32%	52.78%	64.29%

### Comparison of T1-weighted images with CSE-MRI fat fraction maps

**Figure 1:**
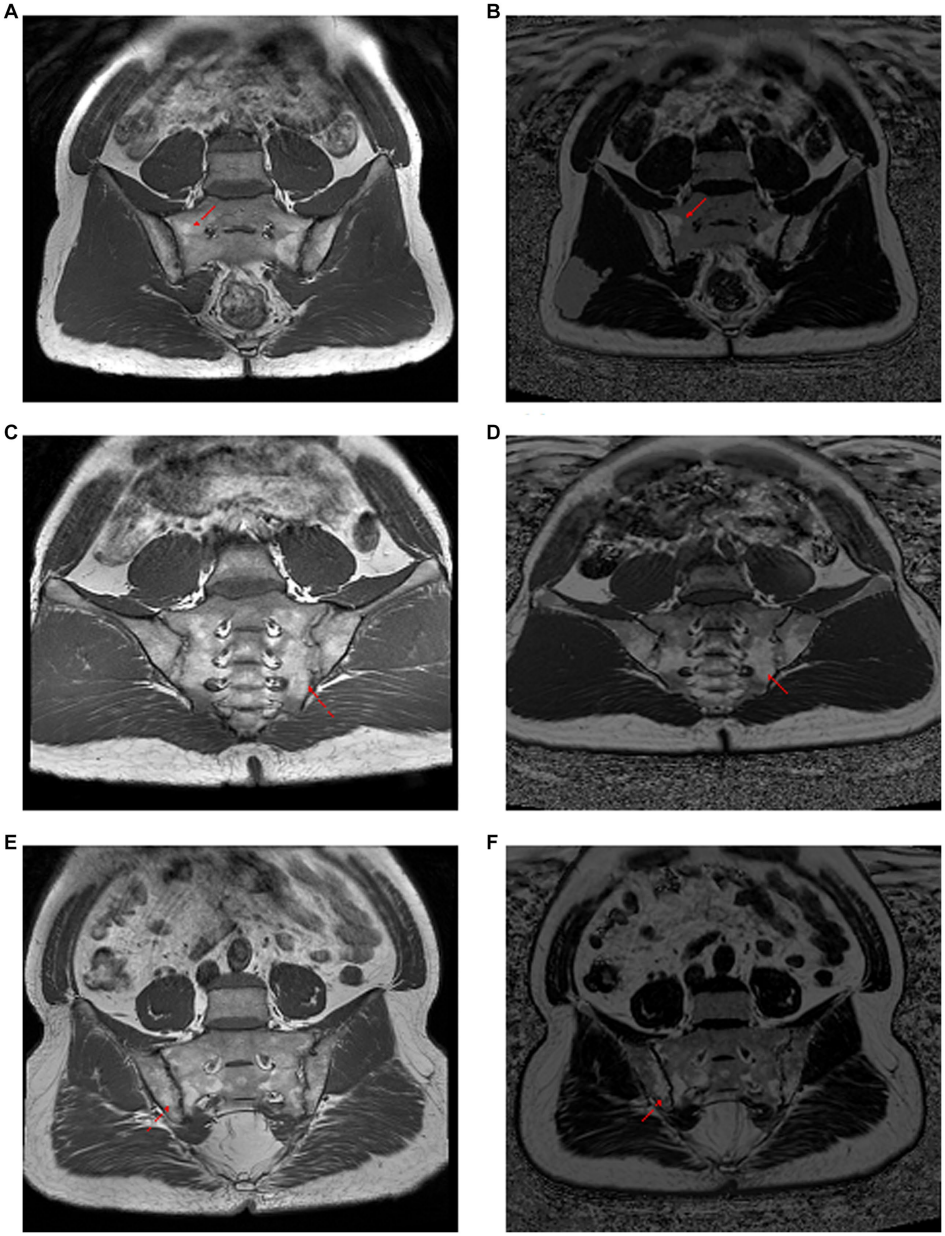
Comparison of T1-weighted images with CSE-MRI fat fraction maps in the observation of fat metaplasia. Panels **(A–F)** were three different ax-SpA patients. Panels **(A,C,E)** are T1-weighted imaging images, and panels **(B,D,F)** are CSE-MRI fat fraction maps. In first and the third patients, the fat deposition pointed by the red arrows are shown as heterogeneous and unclear boundaries on the T1-weighted image, while they are homogeneous and clearly bounded on the CSE-MRI fat fraction map. The second patient had the opposite result. Red arrow: foci of fat deposition.

The difference between fatty lesions on T1-weighted images and CSE-MRI fat fraction maps is shown in Figure 1.

In order to clarify whether there was a difference between T1-weighted images and CSE-MRI fat fraction maps in the recognition of morphological features of fatty lesions, McNemar’s test was applied. Results of the comparisons could be seen in Table 5. No significant difference was found in the distribution of fatty lesions between the two MRI sequences. However, CSE-MRI fat fraction maps could detect significantly more fatty lesions with homogeneity (*p* = 0.0412) and distinct border (*p* = 0.0159) than T1-weighted images in the sacroiliac joint, but not subchondral lesions (*p* = 0.6831).

**Table 5:** Comparison of T1 weighted images and CSE-MRI fat fraction maps in the recognition of anatomic distributions and morphological features of fatty lesions.

	T1(+) CSE-MRI(+)	T1(+) CSE-MRI (−)	T1(−) CSE-MRI(+)	T1(−) CSE-MRI(−)	*P* value
Fat lesion					
At least in 1 quadrant	60	1	3	18	0.6171
At least in 2 quadrants	54	2	4	22	0.6831
At least in 3 quadrants	43	0	1	38	1
At least in 4 quadrants	42	0	1	39	1
Characteristics of fat lesion					
Homogeneity	48	2	4	28	0.6831
Distinct border	40	0	6	36	0.0412*
With at least 1 feature	33	1	10	38	0.0159*
With at least 2 features	52	2	3	25	1
With at least 3 features	40	1	7	34	0.0771
Homogeneity	29	0	10	43	0.0044*

* *p* < 0.05.

Results of the inter-reader agreement of the fatty lesions as well as the morphological features could be seen in Table 6, while the intra-reader agreement could be seen in Supplementary Table S1.

**Table 6:** Inter-reader agreement assessed with Cohen’s Kappa of different fatty lesion features on T1-weighted images and CSE-MRI fat fraction maps.

Feature	T1	CSE-MRI
Presence of fat deposition	0.68 (0.60–0.76)	0.69 (0.61–0.77)
Subchondral location	0.84 (0.75–0.92)	0.91 (0.84–0.97)
Homogeneity	0.80 (0.71–0.89)	0.66 (0.55–0.78)
Distinct border	0.66 (0.54–0.78)	0.75 (0.65–0.85)

## Discussion

Fat deposition *per se* has limited value for nr-axSpA patients so that it should be interpreted in conjunction with other sacroiliac joint lesions to improve the diagnostic accuracy of nr-axSpA (1). We found that there were only 39.13% of nr-axSpA patients had homogeneous or well-circumscribed fatty lesions in the T1-weighted images of nr-axSpA patients, while the feature of being located in subchondral bone could reach 52.17%. The frequency of fat deposition with one feature, two features, and three features were 65.22, 39.13, and 26.09%, respectively. Similarly, Weber et al. found that 20–30% of patients exhibited fatty lesions with three morphological features in the sacroiliac joint.

Noteworthy, fat infiltration could also be observed in the sacroiliac joints in non-SpA patients. In the T1-weighted images, the proportions of non-SpA patients with subchondral, homogeneous and clearly demarcated fatty lesions were 23.81, 9.52 and 14.29%, respectively. Meanwhile, the frequencies of fat deposition accompanied by one feature, two features, and three features were 23.81, 14.29, and 9.52% respectively, which were lower than SpA patients. Further analysis of diagnostic utility showed that the specificities of homogeneity and clear boundaries were the highest, reaching 90.48 and 85.71%, while being subchondral was only 76.19%, which reflected that the diagnostic value of being subchondral is inferior to homogeneity and distinct border.

Our results suggested that more morphological features and more quadrants with fatty lesions could improve the specificity of fat infiltration in axSpA. Fatty lesions in r-axSpA tend to have a higher fat fraction and be more extensive in the sacroiliac joint. This is due to the fact that patients with r-axSpA usually have longer disease durations and more severe sacroiliac joint destruction. Other than number of quadrants, fat deposition in r-axSpA exhibits more morphological features. 55.26% of the r-axSpA patients exhibited fatty lesions with all three morphological features. Based on these results, typical fatty lesions with all three morphological features are much more common in r-axSpA, but relatively rare in nr-axSpA patients, since the structural destruction in nr-axSpA patients tend to be mild. In clinical practice, r-axSpA patients could easily be identified with plain radiographs, since the structural changes in the sacroiliac joints could be conspicuous. Therefore, fatty lesions, even with the typical morphological features, are unlikely to offer much incremental diagnostic value. However, our results did confirm that the three morphological features, especially homogeneity and distinct border, were rather specific to axSpA, albeit less common in nr-axSpA.

The strength of CSE-MRI lies in its ability to quantify fat fractions in the region of interest (7), yet the fat fraction map generated by this sequence could also be a powerful tool to visualize the otherwise inconspicuous morphological features (12). This is the first study to utilize the fat fraction map of CSE-MRI to visualize the morphological features of fatty lesions in axSpA in comparison with traditional T1-weighted images. Although our study failed to prove that CSE-MRI could visualize more fatty lesions, observers could detect more typical morphological features in the fatty lesion, especially the feature of homogeneity and distinct border. Interestingly, such improvement in the observation of morphological features does not incur a loss in the specificity. Therefore, a CSE-MRI fat fraction map could be a helpful addition to the observation of fatty lesions in the sacroiliac joints. CSE-MRI is currently considered the state-of-the-art technique in the quantification of fat signal. Compared with other sequences such as T1-in-and-out-of-phase (IOP) MRI, CSE-MRI is a more sophisticated fat quantification approach which corrects for a number of confounding factors including T1 bias, noise bias and eddy currents. The accuracy of the fat quantification by CSE-MRI could be attributed to such intricate correction calculations, thus generating fat fraction maps enabling better observation of morphological features.

Other than CSE-MRI, a couple of other MRI sequences could also facilitate better characterization of fatty lesions, Dixon sequences rely on the difference in resonance frequency between water and fat, unlike fat suppression sequences. Hence, Dixon sequences could generate water-only, fat-only, in-phase and out-of-phase images in a single acquisition (13, 14). Kuetting et al. used multipoint Dixon technique to non-invasively distinguish chylous and nonchylous ascites and pleural effusions (15). This study showed that fat fraction was greater for chylous versus nonchylous fluids, and *in vivo* fat fraction was correlated with triglyceride content (15). Sequences such as volumetric interpolated breath-hold examination with fat saturation (VIBE-FS) are also promising techniques for the observation of fatty lesions (16, 17). In the future, head-to-head comparisons can be conducted to directly compare different fat-sensitive sequences in the detection and quantification of fatty lesions in SIJ.

It should be pointed out that this study had some limitations. There might be systemic bias in the study design as observers may have overestimated the diagnostic performance of CSE-MRI fat fraction maps in visualizing the morphological features and underestimated in T1-weighted images. To minimize this bias, all T1-weighted images and CSE-MRI fat fraction maps were presented in an independent, random manner, and when there was inconsistency between the two observers, a senior radiologist made the final decision. Another limitation to this study is the relatively small sample size. A cohort of 82 patients with lower back pain may not fully present the potential of CSE-MRI fat fraction maps in the detection of fatty lesions. It would be desirable to include a larger number of patients, as well as acquiring Dixon and VIBE with fat saturation images in the future.

## Conclusion

Fatty lesions with morphological features of being subchondral, homogeneity and distinct border could only be found in 52.17, 39.13 and 39.13% of nr-axSpA patients. Of the three morphological features, homogeneity and distinct border are more specific to axSpA as compared to being subchondral. Typical lesions with all three morphological features could be found in 26.09 and 55.26% of nr-axSpA and r-axSpA patients, yet such lesions could provide a specificity of 90.48%. CSE-MRI fat fraction map is superior to T1-weighted images in the recognition of fatty lesions with homogeneity and distinct border, but could not visualize more fatty lesions.

## Data availability statement

The original contributions presented in the study are included in the article/Supplementary material, further inquiries can be directed to the corresponding authors.

## Ethics statement

The studies involving humans were approved by the Ethics Committee of the Third Affiliated Hospital of Sun Yat-sen University. The studies were conducted in accordance with the local legislation and institutional requirements. The participants provided their written informed consent to participate in this study. Written informed consent was obtained from the individual(s) for the publication of any potentially identifiable images or data included in this article.

## Author contributions

JZ and DL contribution to the design of the work and wrote the manuscript. DL performed the statistical analysis. QC, BL, and CL read images. JG contribution to devised study protocol and approved the submitted version. All authors contributed to the article and approved the submitted version.

## Funding

This work was supported by the grants from the National Natural Science Foundation of China (81871294); the Science and Technology Planning Project of Guangdong Province, China (2019B030316004); Guangdong Clinical Research Center of Immune diseases (2020B1111170008).

## Conflict of interest

The authors declare that the research was conducted in the absence of any commercial or financial relationships that could be construed as a potential conflict of interest.

## Publisher’s note

All claims expressed in this article are solely those of the authors and do not necessarily represent those of their affiliated organizations, or those of the publisher, the editors and the reviewers. Any product that may be evaluated in this article, or claim that may be made by its manufacturer, is not guaranteed or endorsed by the publisher.
