# Supplementary material for: The diagnostic value of morphological features of fat deposition of sacroiliac joint steatosis in axial spondyloarthritis
Source: Front Med (Lausanne). 2023 Aug 23;10:1218834. doi: 10.3389/fmed.2023.1218834 (PMC10484708; doi:10.3389/fmed.2023.1218834)
Supplement: Supplementary file 1 [file Table_1.DOCX]

Table S1 Intra-reader agreement assessed with Cohen’s Kappa of different fatty lesion features on T1-weighted images and CSE-MRI fat fraction maps

| Reader | Feature | T1 | CSE-MRI |
| --- | --- | --- | --- |
| Reader 1 | Presence of fat deposition | 0.88 (0.81-0.96) | 0.9 (0.83-0.97) |
|  | Subchondral location | 1 (1-1) | 0.95 (0.87-1) |
|  | Homogeneity | 0.98 (0.93-1) | 1 (1-1) |
|  | Distinct border | 1 (1-1) | 0.98 (0.93-1) |
| Reader 2 | Presence of fat deposition | 0.66 (0.54-0.77) | 1 (1-1) |
|  | Subchondral location | 0.93 (0.84-1) | 0.87 (0.76-0.98) |
|  | Homogeneity | 0.72 (0.57-0.87) | 0.38 (0.17-0.58) |
|  | Distinct border | 0.55 (0.35-0.74) | 0.73 (0.58-0.88) |
